# Bridging medical education goals and health system outcomes: An instrumental case study of pre-clerkship students’ improvement projects

**DOI:** 10.1007/s40037-022-00711-1

**Published:** 2022-04-08

**Authors:** Bridget C. O’Brien, Josué Zapata, Anna Chang, Edgar Pierluissi

**Affiliations:** 1 Department of Medicine and Education Scientist, Center for Faculty Educators, University of California San Francisco, San Francisco, CA USA; 2 Department of Medicine, University of California San Francisco, San Francisco, CA USA

**Keywords:** Quality improvement, Health systems science, Curriculum, Evaluation

## Abstract

**Introduction:**

Many medical schools engage students in health system improvement (HSI) efforts. Evaluation of these efforts often focuses on students’ learning outcomes and rarely considers the impact on health systems, despite the significant commitment health systems make to these efforts. Our study identified and evaluated system-level outcomes of pre-clerkship medical students’ engagement in HSI efforts.

**Methods:**

We used an instrumental case study approach to examine the effects of pre-clerkship medical students’ engagement in HSI projects as part of a 15-month experiential curriculum. We extracted data from 53 project summaries and posters completed during the 2017–18 academic year and follow-up survey data collected in May 2019 from physician coaches and health system professionals who mentored students, contributed to these projects, and worked in the clinical microsystems where the projects occurred.

**Results:**

We identified three categories and ten indicators of health system outcomes relevant to medical student engagement in HSI. Using these indicators, our evaluation found multiple benefits to the microsystems in which projects occurred. These included achievement of project aims, perceived immediate and sustained project impact on the health system, and development and implementation of projects with aims that aligned with national and health system priorities.

**Conclusion:**

Evaluation of HSI curricula needs to include effects on health systems so that program design can optimize the experience for all involved. Our study offers a framework others can use to evaluate system-level effects of project-based HSI curricula and shows several ways in which students’ engagement can add value to health systems.

**Supplementary Information:**

The online version of this article (10.1007/s40037-022-00711-1) contains supplementary material, which is available to authorized users.

## Introduction

Healthcare systems face many challenges that medical schools and graduate medical education programs must help address [1–3]. The inclusion of systems-based practice as one of six Accreditation Council for Graduate Medical Education (ACGME) core competencies for physicians in 1999 signalled a commitment to make medical education part of the solution to gaps in healthcare quality, disparities, and value [4, 5]. Educators have identified similar competencies for medical students [6, 7] and proposed health systems science as an overarching framework that covers “the methods and principles for improving quality, outcomes, and costs of healthcare delivery for patients and populations within medical care systems” [8, p. 123–124]. While these efforts represent important strides toward defining desired learning outcomes that can be used to evaluate curricular success from an educational standpoint, the indicators of success from a health systems standpoint are less clear. If medical education aspires to be part of the solution, greater clarity is needed about ways in which educational initiatives may positively affect health systems. In this paper, we explore the system-level effects of an effort to engage medical students in health systems improvement prior to clerkships.

In 2013, the American Medical Association launched “Accelerating Change in Medical Education,” an initiative that challenged medical schools to design curricula that prepare physicians capable of navigating and improving health systems to benefit patients and communities [9]. The approaches to teaching health systems science vary among medical schools [10], but a common guiding principle is to develop students’ competency in health systems science through authentic experiences while helping health systems achieve quality and value goals [11–13]. Health systems improvement (HSI), or quality improvement, involves “identifying, analyzing, or implementing changes in … the healthcare system to improve the performance of any component of the healthcare system” [8, p. 125]. Many medical schools have partnered with health systems to engage learners in improvement efforts [9, 10, 14, 15]. These partnerships are compelling from an educational standpoint as they provide opportunities to develop key HSI competencies (e.g., quality improvement principles; data and measurement; innovation and scholarship) [16] through workplace-based experiential learning, which is widely recognized as an effective means of education [17–19].

The New World Kirkpatrick Model [20] defines four levels of outcomes in medical education: Level 1) reaction—learner satisfaction and engagement; Level 2) learning—modification of attitudes, knowledge and skills; Level 3) behaviour—application of learning to real-world practices; and Level 4) results—changes in patient care, new or improved organizational practices or performance, societal change [20–22]. In the evaluation of undergraduate medical education curricula, level 4 is often omitted because it is resource-intensive to measure and challenging to link directly to medical students’ actions [21–23]. Yet, the activity of students as part of an interdisciplinary HSI team provides an opportunity to include measurement of structure, process, and patient outcomes as well as perspectives of key health system stakeholders [24]. HSI often generates change on longer timeframes, making intermediary outcomes such as stakeholders’ perceptions critical to sustaining the partnership between medical education and health systems that invite student involvement in systems improvement. Without stakeholder buy-in, the commitment of human and other resources from partnering health systems is at risk. These types of level 4 outcomes, from the systems perspective, have rarely been explored. Our study aims to define and evaluate system-level outcomes of students’ engagement in HSI (stakeholder perspectives, structure, process, patient outcomes) in the hope of generating a framework that can guide the evaluation of experiential HSI curricula.

## Methods

### Context

All medical students in their first 15 months at the University of California San Francisco (UCSF) participate in an experiential HSI curriculum as part of the Clinical Microsystem Clerkship (CMC) [25, 26]. The clinical microsystems are housed within one of three health systems (academic, safety net, and veterans’ affairs). Student teams work with a physician coach and staff from the clinical microsystem on a project that addresses a health system problem that is identified prior to students’ arrival. These problems and projects are selected through criteria developed by CMC curriculum directors with input from health system leaders. Key criteria include alignment with national/institutional goals, involvement of interprofessional team members, interaction with patients, consideration of equity and disparities, feasibility to implement over 15 months, and data available to inform the project. This curriculum allows students to learn health systems science through real-world application of concepts [17, 18] while advancing health system goals [11].

Within the HSI curriculum, students are expected to apply Lean A3 improvement methodology [27] to a current health system problem. Students work with health professionals in the microsystem to articulate the problem their project will address, define improvement goals, perform a gap analysis, implement interventions, evaluate outcomes, and reflect on the learning process. The curriculum includes lectures, workshops, and projects. Each group receives guidance from a physician coach and health system QI team members. Student groups submit four interim project summaries and present their completed work as posters to education deans and health system leaders. Our study was reviewed and granted exempt status by our institution’s committee for human research (File number: #19-27272).

### Design

We selected an instrumental case study approach [28], which is the study of a specific example to provide insight into a more general issue or phenomenon. In our study, the case is one academic year of the experiential, project-based portion of the UCSF HSI curriculum and the issue is how to incorporate health system-level outcomes into the evaluation of experiential HSI curricula. We drew on the New World Kirkpatrick Model [20], focusing on level 4 (results) outcomes that could bridge educational and health systems goals of students’ engagement in HSI efforts. Through a combination of literature review, conversations with local health system leaders about essential criteria for students’ HSI projects from the health system perspective, and consideration of available data, we grouped systems-level outcomes of interest into three categories: 1. Project goal completion, 2. Effects on the microsystem, and 3. Project alignment with health system (see Resource 1 of the Electronic Supplementary Material (ESM)).

### Data sources and data collection

We included all projects completed by medical students during the second year the CMC existed at our institution (August 2017 through November 2018). We chose this year to capture effects of improvements made to stabilize the curriculum after the launch year. Also, at the point of data collection, several months had passed since students had completed the CMC, which allowed us to find out about downstream effects of their efforts.

We analyzed data collected from three sources (see ESM resource 2): *Project summaries *completed by student teams using an A3 format to document the steps of their project [29], *Project posters* presented at health system quality improvement (QI) forums, and a *Survey of physician coaches and health system QI project leader* (QI leads) (ESM resource 3). We chose to survey key health system stakeholders rather than institutional leadership because of their direct experience with the HSI curriculum and knowledge of how students’ efforts affected the microsystem [25, 30]. We used existing literature [24, 31] and information extracted from project summaries and posters to develop the survey. Questions asked respondents to describe changes in the microsystem due to the project, to rate the projects’ effect on the microsystem, to state whether effects were sustained, and to identify reasons for these ratings. We piloted the questionnaire by conducting cognitive interviews with two physician coaches and modified questions to enhance clarity based on the feedback received [32]. The final version of the questionnaire was uploaded to Qualtrics, a web-based survey platform, and one team member (JZ) sent personal invitations to each faculty coach and QI lead to participate.

We created a data extraction form to collect descriptive information about project characteristics, aims, interventions, data/metrics, achievement of aims, and barriers/facilitators to project success from project summaries and posters (ESM resource 4). We coded project aims according to whether the primary aim was to change structural, process, or patient outcome measures (see ESM resource 1 for a description). All authors independently tested the extraction form and as a group agreed to a final version. After finalizing the form, we worked in pairs to extract data from all 53 projects. JZ coded all 53 project summaries and posters, the other three investigators coded one-third. Coding discrepancies were reconciled to consensus. Data were recorded in Microsoft excel.

### Data analysis

We calculated descriptive statistics for all categorical and numeric data. Since some projects received multiple survey responses from coaches and QI leads, we aggregated data and used the mean response per project. We coded free text responses to identify categories and themes [33].

## Results

Second year medical students (*n* = 152) completed 53 projects that spanned a broad range of clinical microsystems across three affiliated health systems, five types of clinical settings, and 16 medical sub-specialties. Tab. 1 describes the key characteristics of the projects, Box 1 shows a sample project; a description of two further sample projects can be found in the ESM Box and ESM resource 5 lists all the projects. Twenty-six coaches (96%) and 13 QI leads (68%) completed follow-up surveys for 49 of the projects (92%) (ESM resource 3). We organized our findings into three categories of system-level outcomes with multiple indicators for each category. The first category represents immediate outcomes (accomplishment of project goals), the second captures effects after the project ended, and the third describes project alignment with health system priorities and processes.

**Table 1:** Key features of medical students’ clinical microsystem clerkship health systems improvement projects, 2017–18

Key features	No. projects (% of 53)
*Health system*	
Academic	22 (42)
Safety net	19 (36)
Veterans affairs	12 (23)
*Clinical microsystem type*	
Adult medicine	48 (91)
Pediatric medicine	5 (9)
Ambulatory care	27 (51)
Acute care	18 (34)
Surgical/perioperative care	7 (13)
Other	1 (2)
*Project aims to improve … (IOM priorities*^*34*^* projects may address multiple) (Indicator 3.1)*	
Effectiveness	41 (77)
Safety	18 (34)
Patient-centeredness	10 (19)
Efficiency	9 (17)
Equity	8 (15)
Timeliness	5 (9)
*Type of measure (Indicator 2.3)*	
Structural change	1 (2)
Process change	36 (68)
Patient outcome	16 (30)
*Project achieved at least 1 aim (based on data provided in project summary report or poster) (Indicators 1.1 and 2.3)*	
Yes	28 (53)
– Structural changes	1
– Process changes	19
– Patient outcomes	8
No	24 (45)
– Process changes	16
– Patient outcomes	8
Could not determine	1 (2)
– Process changes	1

### Box 1 Description of a sample project

**Improving hypertension control in black patients at an academic primary care clinic**

This project focused on reducing a healthcare disparity in hypertension control in a primary care clinic. At baseline, the rate of uncontrolled hypertension among Black patients in this primary care clinic was significantly higher (35%) than that of patients of all other races (26%). The student team set a goal of closing this gap and reducing the rate of uncontrolled hypertension among Black patients to that of patients of all other races within six months. To explore potential reasons for this disparity, students performed a literature review, interviewed nursing staff, the clinic practice manager, and patients. They learned that the underlying causes varied from patient to patient. They designed an intervention in which a student on the team called each Black patient with uncontrolled hypertension to discuss the patient’s understanding of hypertension, hear their concerns about their ability to manage this condition, and schedule follow-up clinic appointments, if appropriate. Over the course of the project, the rate of uncontrolled hypertension among Black patients decreased from 35% to 30%, short of the team’s stated goal of 26%. However, the project increased utilization of blood pressure appointments with nurses which were often more convenient for patients. It also initiated a change across the health system to the electronic medical record so that all office visit blood pressure measurements are utilized in determining hypertension control status, not just those from primary care. At the end of the students’ curricular time, the effort was taken over by clinic staff and the scope increased to encompass all primary care clinics within the academic medical center. The clinic QI lead reported that this project improved their microsystem, commenting that “embedded telephone outreach and follow-up workflow is now being developed, in large part due to the enthusiasm and results from the students’ intervention.” Additionally, the students achieved learning goals in the areas of quality improvement and health disparities.

### Category 1: Project goals accomplished by end of the curriculum

Indicator 1.1 Achievement of project aims: More than half (53%) of the project summaries and posters indicated achievement of at least one aim (Tab. 1).

Indicator 1.2 Achievement of educational goals: Survey data indicated that most physician coaches and QI leads (86%) agreed that students achieved the primary educational goal of applying HSI principles. Respondents commented on students’ appreciation of the complexities of quality improvement, valuing patients and interprofessional colleagues as key stakeholders in the improvement process, applying the steps of Lean methodology through a hands-on experience, conducting outcome measurement from Plan-Do-Study-Act (PDSA) cycles, and presentation skills. These findings suggest that members of the health system felt that students learned to apply HSI knowledge and skills, a potentially important health system goal since many students will go on to train and work in these settings.

### Category 2: Effects of the HSI project on the system

Indicator 2.1 Perceived effects at the end of the curriculum (15 months): Based on coach and QI lead survey responses, many projects (67%) had a moderate or substantial impact on the microsystem at the time students completed their involvement with the project (Tab. 2)—even if project aims were not achieved. For projects perceived as having minimal or no effect on the microsystem, reasons included structural or bureaucratic barriers (e.g. difficulty getting intervention approved), insufficient time for students to complete the project (e.g. poor project management, unrealistic scope, or schedule conflicts that limited participation in key activities), and project completed or adopted by another group separate from students’ efforts.

**Table 2:** Perceived impact of medical students’ clinical microsystem clerkship health systems improvement projects from 2017–18 based on follow-up survey responses from coaches and QI leads (collected in May 2019)

Survey responses and sample explanations from free response	No. projects (% of 49^a^)
**Indicator 2.1 Perceived impact at project end (15 months)**	
***Impact of project on microsystem at time students left (approx. Nov 2018)***	
None (e.g., did not meet proposed goal)	3 (6)
Minimal (e.g., project part of larger effort, hard to connect outcomes to students’ efforts, persistent structural barriers)	13 (27)
Moderate (e.g., students sparked interest that increased outreach efforts, pilot phase)	23 (47)
Substantial (e.g., improvement in valued metrics such as documentation, screening, illuminated a problem that was then addressed, provided a framework or foundation for future efforts)	10 (20)
**Indicator 2.2 Perceived lasting impact of project (7-month post-curriculum)**	
***Did the project have impact after the students left the microsystem? (approx. May 2019)***	
*Yes because …*	**37/49 (76)**
– Project (including all, some piece of it, or next phase) was taken on by health system staff	26/37 (70)
– Project has been ‘hardwired’ into the microsystem, requires minimal effort to sustain	16/37 (43)
– Project (including all, some piece of it, or next phase) was taken on by a new cohort of students	3/37 (8)
– Other (e.g., educational efforts help sustain change; culture shift among key health professionals in ways that support the intervention; identified a need for outside consultants who then had a huge impact)	5/37 (14)
*No because …*	**12/49 (25)**
– When the students left there was no one to do the work	6/12 (50)
– Project was no longer relevant (circumstances/priorities changed)	3/12 (25)
– Project identified other areas that needed to be prioritized	2/12 (17)
– Other (e.g., lack of buy in from key health professionals)	5/12 (42)

^a^ Survey responses received from coaches and/or QI leads of 49 out of 53 projects. Multiple responses for the same project were aggregated.

Indicator 2.2 Perceived effects post-curriculum (7 months post-curriculum): Based on coach and QI lead survey responses, many projects (76%) had a sustained impact on the microsystem seven months later (Tab. 2). One respondent described how student engagement could inspire subsequent efforts, *“Student work sparked interest in focusing specifically on hypertension control for African Americans in the broader primary care organization. Outreach efforts were expanded.” *Coaches and QI leads attributed sustainability to health system staff continuing the improvement work and/or incorporation of interventions into usual processes. The most commonly cited reasons projects were not sustained included a shift in health system priorities, and lack of resources to continue the work without students, as one respondent wrote, “*There was no one to push to work forward after the students left and the project fell by the wayside.”*

Indicator 2.3 Structures, processes, and patient outcomes: Projects focused on changing structures (1 project), processes (36 projects), or patient outcomes (16 projects). One project made a structural change by producing a handout about delirium for family members of patients in the intensive care unit. Of the 36 projects focused on process changes, 19 (53%) achieved their primary aim. For example, the depression screening rate in a primary care clinic increased from 58% to 63%, the median time from door to thrombolytic infusion in patients with stroke decreased from 38 to 30 min, and the proportion of psychiatric inpatients attending a first outpatient mental appointment increased from 55% to 64%. Of the 16 projects focused on changing a patient outcome measure, 8 (50%) achieved their primary aim. For example, the rate of in-hospital injury from falls decreased from 0.58 to 0.52 per 1000 patients and influenza vaccination rates increased from 37 to 58% in a primary care clinic.

Indicator 2.4 Balance of costs and benefits: While our data did not allow a true cost-benefit analysis, information about barriers and facilitators to project success gave us insight into some required resources or investments from the system to support the HSI projects. These included staff time to meet with students, participate in the design and pilot implementation of interventions, as well as helping students navigate the microsystem (e.g., accessing data and connecting them with key stakeholders and local champions). Additional time for coaches and QI leads to assist with project management was also important.

The benefits described by coaches and QI leads largely focused on the educational value to students such as learning and experiencing *“the steps of QI work,” “how to work with a multidisciplinary team,” *and* “the crucial role of getting buy-in from all stakeholders.”* While some coaches and QI leads highlighted small but valuable improvements from students efforts, such as raising awareness of gaps in care, introducing processes that helped streamline and standardize care, figuring out why a proposed intervention might not work and *“improving the quality of the (patient satisfaction) tool and testing workflow for its administration”,* others felt it was difficult or even unrealistic *“for students to truly improve a system with the amount of time they have.”* Nonetheless, many saw the value of the system long-term, noting *“if they get an exposure to Lean and design thinking and QI methods, that is itself a win.”*

### Category 3: Project alignment with health system priorities and processes

Indicator 3.1 Alignment with health system or microsystem goals: From our analysis of all three data sources, we found many signs of project alignment with health system priorities. We used Institute of Medicine (IOM) priorities [34] as all three health systems embraced these. All projects addressed one or more IOM priority area; improving the effectiveness of care was most common (Tab. 1). Most projects also addressed a national and/or local priority (77%). Nearly half referenced a published benchmark (45%). Addressing a healthcare disparity was an explicit goal in 15% of projects.

Indicator 3.2 Involvement of key stakeholders: Analysis of project reports and posters showed that all student teams engaged clinicians from multiple health professions in one or more core improvement step. Professions included nursing, pharmacy, physical therapy, psychology, social work, and data science as well as administrative and research personnel. Twenty-three projects (43%) involved direct interactions between students and patients or families to understand the gap or to design, implement, or measure the intervention. Projects that did not involve direct interaction with patients focused on interventions for clinicians (e.g., note templates or clinician training). These findings suggest success in engaging key stakeholders from clinical microsystems in students’ HSI projects.

Indicator 3.3 Use of health system improvement tools and interventions: We found that projects used tools familiar to the health systems in which the projects occurred. Most projects (87%) used fishbone diagrams to represent their gap analysis. Others used process maps, Possible-Implement-Challenge-Kill (PICK) charts, and five why’s [35, 36]^.^ This finding suggests alignment between the language, concepts and tools students learned in the formal medical school curriculum and the language, concepts and tools used in the clinical microsystems. Projects often included more than one intervention (74%) reflecting sensitivity to the need for multi-pronged approaches. Almost all projects (89%) attempted to provide in-process support (e.g. team huddles, or electronic health record templates, alerts, or checklists) that could enhance existing processes in the microsystem. Other common interventions included training and education for healthcare professionals, staff, patients, and/or family members (72%) and audit and feedback (26%).

Indicator 3.4 Reasonable scope for implementation: Most projects (79%) fully implemented at least one intended intervention within the timeframe of the curriculum and 19% partially implemented at least one intervention. All but one project completed at least one PDSA cycle. The average duration of the intervention period was five months. These findings suggest that projects could be designed and managed to support completion of specific goals and activities within the expected 15 month period.

## Discussion

As medical schools engage students in health systems improvement, the effects of such curricula need to be defined in ways that address the goals of stakeholders in both the educational and health systems enterprise. The New World Kirkpatrick model [20] is widely used to evaluate learning outcomes at the individual level, with less attention to system-level outcomes. We identified and used three general outcome categories and ten specific indicators to evaluate the clinical microsystem-level effects of a project-based HSI curriculum (Kirkpatrick level 4). In our study, 30% of projects addressed patient outcomes and nearly half of these achieved their aims. We found that two-thirds of projects were perceived to have moderate or substantial impact on the microsystem, often with effects that extended beyond students’ time in the curriculum. While these effects did not always correspond to achievement of projects’ specific aims or targeted metrics, other types of benefits accrued such as new insight into a problem, staff education or heightened awareness of an issue, or recognition of reasons why a proposed intervention would not work. While projects required resources, they generally aligned with health system priorities and processes. Based on our findings, we discuss implications of our work for future efforts to evaluate project-based HSI curricula.

Our study adds to recent literature that seeks to understand how students can add value to both individuals and systems for healthcare organizations that offer clinical placements to trainees [37–39]. Two reviews [37, 38] focused on individual-level effects on health system educators and suggested that the benefits educators experience when working with students (e.g., enjoyment of teaching, opportunity to gain new knowledge and improve skills) may outweigh costs (e.g., increased workload, decreased productivity). We did not explicitly collect data about perceived benefits and burdens among staff who worked with students in the microsystem, though some project summaries and survey comments on barriers, facilitators and perceived impact addressed this. In retrospect we see this information as an important component of value to health systems and recommend including it in cost-benefit indicators.

Few studies have explored organizational level effects of student engagement in systems improvement [39]. Based on interviews with placement educators, Kemp and colleagues [39] found that students were perceived as adding to workforce capacity by moving valued projects forward, creating ‘physical deliverables’ such as resources and videos, creating new partnerships and launching new projects. Challenges included costs such as time invested in planning for and supervising students and working with students who lacked necessary professional skills to complete tasks independently. The authors found that over time, the organizational benefits increased and costs decreased with more trust and stronger partnership between the organizations and the university. These findings underscore the importance of including system-level indicators as part of routine evaluation so effects can be monitored over time and experiences that could diminish trust between medical schools and clinical microsystem partners can be addressed.

Our findings also illuminate other challenges encountered by students, coaches and staff. Some of these challenges such as project scope, access to data, and schedule conflicts may be anticipated and provide useful guidance for future project planning. Other challenges such shifting organizational priorities, loss of key personnel, and delays in implementing changes to the electronic medical record may be harder to anticipate.

Prior work has suggested value-added roles for students [11], but to our knowledge has not followed up to examine the perceived or actual value of students’ contributions to clinical microsystems. One reason may be that value is often conceptualized in monetary terms, yet many of the valued outcomes are difficult to monetize—particularly in a way that is feasible for routine program evaluation. Our study offers a way to conceptualize value in broader terms based on academic products (project summaries, posters) and stakeholder perceptions. Subsequent work may benefit from including a broader array of stakeholders such as additional staff, patients, and administrators in the health system whose work or experience have been affected by the students’ efforts.

### Limitations

We derived categories and indicators for microsystem-level outcomes based in part on the HSI curriculum and data available at our institution, which may limit the transferability of indicators to other contexts. Nonetheless, the three general categories can be used as a starting point for identifying indicators specific to different settings. We collected data from sources that may emphasize positive outcomes and under-report critiques, challenges, or unsuccessful aspects of the experience. While survey respondents may have been reluctant to say the projects did not have an impact, the fact that some admitted challenges and explained reasons gives us some encouragement about the credibility of the data. Additional sources of information could be sought beyond the coaches and QI leads, perhaps from external reviewers who were less directly involved in projects, to gauge impact.

## Conclusion

As project-based health systems improvement curricula become more common in medical student education, comprehensive evaluation plans that include realistic, meaningful indicators of system-level outcomes along with student learning outcomes will provide critical information to sustain strong partnerships between medical schools and health systems. Our study demonstrates the value of including the perspectives of health system physicians and clinical staff who observe the effects of students’ contributions first-hand, as well as their impact on the clinical microsystem after the curriculum ends. Through such engagement of key stakeholders, these curricula can be further adapted to drive outcomes that matter not just to students and educators, but also to health systems leaders and to the patients that these systems serve.

## Supplementary Information

Resource 1. Systems-level Outcome Categories, Indicators, Descriptions, and Example Data Sources to Evaluate Student Engagement in Health Systems Improvement Projects

Resource 2. Description of Data Used to Identify Outcomes of Interest to Health Systems Stakeholders from Medical Students’ Clinical Microsystem Clerkship (CMC) Health Systems Improvement Projects, 2017–2018

Resource 3. Survey of faculty coaches and QI leads to follow up on Clinical Microsystem Clerkship (CMC) health systems improvement project status

Resource 4. Data Extraction form for HSI Project Summaries and Posters

Resource 5. List of 2017–18 University of California, San Francisco Clinical Microsystem Clerkship health system improvement projects, organized by clinical setting and specialty

Box. Sample project descriptions
