# Supplementary material for: Bridging medical education goals and health system outcomes: An instrumental case study of pre-clerkship students’ improvement projects
Source: Perspect Med Educ. 2022 Apr 8;11(4):179–86. doi: 10.1007/s40037-022-00711-1 (PMC9391531; doi:10.1007/s40037-022-00711-1)
Supplement: Supplementary file 1 — Resource 1. Systems-level Outcome Categories, Indicators, Descriptions, and Example Data Sources to Evaluate Student Engagement in Health Systems Improvement Projects [file 40037_2022_711_MOESM1_ESM.docx]

ESR 1.

**Systems-level Outcome Categories, Indicators, Descriptions, and Example Data Sources to Evaluate Student Engagement in Health Systems Improvement Projects**

| **Outcome Categories and Indicators** | **Description** | **Example Data Source** (from UCSF case study) |
| --- | --- | --- |
| **Category 1: Project goals accomplished by end of curriculum**  *Description: Project goals include specific aims of the project and educational goals or learning objectives associated with the project*  Data Source: Data extracted from project summaries and posters; Survey data from microsystem stakeholders | | |
| Indicator 1.1 Achievement of project aims | Whether or not the specific aims of the project are met | SMART goals and final outcomes reported for each goal |
| Indicator 1.2 Achievement of educational goals | Staff members involved in the health systems improvement (HSI) project perceive students to have learned the principles and practice of HSI through the project | Rating of students’ perceived learning of HSI principles; Written comments describing what students learned |
| **Category 2: Effects of the HSI project on the microsystem**  *Description: Key stakeholders in the microsystem describe their observations and experiences of the projects’ effects on outcomes they value*  Data Source: Data extracted from project summaries and posters; Survey data from microsystem stakeholders | | |
| Indicator 2.1 Perceived effects at the end of the curriculum | Staff members’ perceptions of the value and impact of the project at the end of the curriculum / students’ involvement in the project | Rating of magnitude of project effect at end of curriculum and description of effect; Rating of perceived value to microsystem |
| Indicator 2.2 Perceived effects post-curriculum | Staff members’ perceptions of the value and impact of the project several months after the end of the curriculum / students’ involvement – sustainability and sustained effects of the project | Status of project at 7-month follow up, written comments explaining effects and contributing factors |
| Indicator 2.3 Structures, processes, and patient outcomes | Project addresses structure, process, or patient outcome measures based on definitions from Donabedian 1988^a^: Structures are “attributes of the settings in which care occurs.” Processes are “what is actually done when giving and receiving care.” Outcomes are “the effects of care in the health status of patients and populations.” | SMART goals and final outcomes reported for each goal |
| Indicator 2.4 Balance of costs and benefits | Monetary value of costs and benefits are difficult to calculate directly. Perceived or actual resource use can be compared to perceived or actual benefits to check for alignment and sources of discrepancy | Proxies for actual costs and benefits; Description of barriers and facilitators (which require resources and represent costs); Reported project outcomes (benefits) |
| **Category 3: Project alignment with health system priorities and processes**  *Description: Project is designed and conducted in a way that is consistent with stated priority areas embraced by the health system and quality/system improvement frameworks and methods used in the health system*  Data Source: Data extracted from project summaries and posters | | |
| Indicator 3.1: Alignment with health system or microsystem goals | Project addresses national, regional and/or local clinical priorities and goals | Project aims address Institute of Medicine (IOM) and/or other national or local priorities |
| Indicator 3.2 Involvement of key stakeholders | Project represents input from staff, patients, and families who will be directly involved in implementing or receiving the intervention and/or contributing to project design | Gap analysis, intervention, and/or implementation involves:   - staff members who work in the clinical microsystem, or   patients and families served by the clinical microsystem |
| Indicator 3.3: Use of health system improvement tools and interventions | Quality improvement (QI) methodology, tools, and language used by students match those used by health system stakeholders; Interventions reflect consideration of needs, resources, and integration with current workflow | Gap analysis and intervention selection includes Lean improvement tools; Interventions align with microsystem needs, capacity, and workflow |
| Indicator 3.4: Reasonable scope for implementation | Proposed interventions are implemented within allotted timeframe; Students participate in implementation | Implementation of at least one intervention and completion of at least one Plan-Do-Study-Act (PDSA) cycle in 15 months; Lessons learned |

^a^ Donabedian A. The Quality of Care: How Can It Be Assessed? JAMA. 1988;260(12):1743–1748.
