# Supplementary material for: Bridging medical education goals and health system outcomes: An instrumental case study of pre-clerkship students’ improvement projects
Source: Perspect Med Educ. 2022 Apr 8;11(4):179–86. doi: 10.1007/s40037-022-00711-1 (PMC9391531; doi:10.1007/s40037-022-00711-1)
Supplement: Supplementary file 2 — Resource 2. Description of Data Used to Identify Outcomes of Interest to Health Systems Stakeholders from Medical Students’ Clinical Microsystem Clerkship (CMC) Health Systems Improvement Projects, 2017–2018 [file 40037_2022_711_MOESM2_ESM.docx]

**ESR 2.**

**Description of Data Used to Identify Outcomes of Interest to Health Systems Stakeholders from Medical Students’ Clinical Microsystem Clerkship (CMC) Health Systems Improvement Projects, 2017-2018**

| **Data Source** | **Description** | **Data Collected for Analysis** |
| --- | --- | --- |
| **Project summaries** | - A structured template based on the Lean A3 framework^29^ in which student teams document the steps of their project - Includes seven sections: background; current conditions; target conditions (goals); gap analysis; experiments; action plan; study, reflect, plan next steps - Each student team submits a complete project summary at end of the CMC | Key characteristics of projects:   - Aims - Interventions selected and implemented - Reported outcomes (aim fully, partially or not achieved) - Barriers and facilitators to aim achievement |
| **Project Posters** | - A course requirement that describes the team’s system improvement project - Presented at local health system quality improvement forums | - Interprofessional interactions - Updated outcome information (after project summary submitted) - Additional barriers and/or facilitators |
| **Survey of faculty coaches and QI leads^a,b^**  (Appendix 1) | - 13-item online survey administered through Qualtrics eight months after the curriculum ended - Faculty coaches and QI leads received an email invitation to participate in May 2019 and received up to three reminders - Questions asked about:   - the status of each project at the end of the curriculum and eight months later (multiple choice and free text)   - factors contributing to the current status of the project (multiple choice and free text)   - Overall success of the project for microsystem improvement and student learning | Faculty coach and QI lead perceptions of:   - Aim achievement - Immediate and sustained project impact on microsystem - Factors contributing and inhibiting project outcomes and impact - Success of the project in achieving two objectives: improving clinical microsystems and supporting students’ learning of HSI |

^a^ QI Leads are physicians or staff members from the microsystem who guided the project along with the faculty coach

^b^ Some coaches worked with two or three student teams and completed the survey for each project

**Table 3. Key Features of Medical Students’ Clinical Microsystem Clerkship Health Systems Improvement Projects, 2017-18**

| **Key Features** | **No. Projects**  **(% of 53)** |
| --- | --- |
| Health System | |
| Academic | 22 (42) |
| Safety Net | 19 (36) |
| Veterans Affairs | 12 (23) |
|  |  |
| Clinical Microsystem Type | |
| Adult Medicine | 48 (91) |
| Pediatric Medicine | 5 (9) |
|  |  |
| Ambulatory Care | 27 (51) |
| Acute Care | 18 (34) |
| Surgical/Perioperative Care | 7 (13) |
| Other | 1 (2) |
|  |  |
| Project Aims to Improve… (IOM priorities^35^, projects may address multiple) | |
| Effectiveness | 41 (77) |
| Safety | 18 (34) |
| Patient-Centeredness | 10 (19) |
| Efficiency | 9 (17) |
| Equity | 8 (15) |
| Timeliness | 5 (9) |
|  |  |
| Project Achieved at Least 1 Aim (based on data provided in project summary report or poster) | |
| Yes | 28 (53) |
| No | 24 (45) |
| Could Not Determine | 1 (2) |

**Table 4. Perceived Impact of Medical Students’ Clinical Microsystem Clerkship Health Systems Improvement Projects from 2017-18 based on Follow-Up Survey Responses from Coaches and QI Leads (collected in May 2019)**

| **Survey Responses and Sample Explanations from Free Response** | **No. Projects**  **(% of 49^a^)** |
| --- | --- |
| **Perceived Impact at Project End (15 months)** | |
| ***Impact of project on microsystem at time students left (approx. Nov 2018)*** | |
| None (*e.g., did not meet proposed goal)* | 3 (6) |
| Minimal (*e.g., project part of larger effort, hard to connect outcomes to students’ efforts, persistent structural barriers)* | 13 (27) |
| Moderate *(e.g., students sparked interest that increased outreach efforts, pilot phase)* | 23 (47) |
| Substantial *(e.g., improvement in valued metrics such as documentation, screening, illuminated a problem that was then addressed, provided a framework or foundation for future efforts)* | 10 (20) |
|  |  |
| **Perceived Lasting Impact of Project (7-month post-curriculum)** | |
| ***Did the project have impact after the students left the microsystem? (approx. May 2019)*** | |
| **Yes because …** | **37/49 (76)** |
| - Project (including all, some piece of it, or next phase) was taken on by health system staff | 26/37 (70) |
| - Project has been ‘hardwired’ into the microsystem, requires minimal effort to sustain | 16/37 (43) |
| - Project (including all, some piece of it, or next phase) was taken on by a new cohort of students | 3/37 (8) |
| - Other (e.g., educational efforts help sustain change; culture shift among key health professionals in ways that support the intervention; identified a need for outside consultants who then had a huge impact) | 5/37 (14) |
| **No because…** | **12/49 (25)** |
| - When the students left there was no one to do the work | 6/12 (50) |
| - Project was no longer relevant (circumstances / priorities changed) | 3/12 (25) |
| - Project identified other areas that needed to be prioritized | 2/12 (17) |
| - Other (e.g., lack of buy in from key health professionals) | 5/12 (42) |
|  |  |

^a.^ Survey responses received from Coaches and/or QI Leads of 49 out of 53 projects. Multiple responses for the same project were aggregated.

**Box 1: Three Sample Project Descriptions**

**Reducing Pregnancy-Related Deaths from Obstetric Hemorrhage at a County Hospital**

This project focused on reducing obstetric mortality by implementing a new, state-wide, institutional performance metric at a County Hospital. This project aimed to implement an evidence-based, quantitative blood loss (QBL) measurement process for 80% or more births each year (the state-wide benchmark) from a current baseline of zero. Based on a literature review, interviews, focus groups, and surveys of midwives, nurses and physicians, students identified the lack of protocols, training, and necessary equipment as important barriers to address. They partnered with quality improvement leaders to develop a standard protocol for QBL assessment, delivered an in-person and online training for providers, and obtained a dedicated scale to facilitate measurement of QBL by weighing linens. After implementation, the team performed audits of births and provided feedback to reinforce the importance of QBL assessment. This team exceeded the project aim, achieving average weekly compliance with QBL techniques in 84% of deliveries within nine months. Surveys of clinic staff at the conclusion of the project characterized the degree of improvement as “substantial,” commenting that “[the] blood loss calculation system had been completely changed to quantitative compared to estimated” as a result of the student project. Additionally, students reported learning how to incorporate medical literature in clinical practice, the importance of understanding workflow when designing systems changes, and the need to involve all stakeholders when developing interventions. The QBL process became the new “norm” and continued after the students left the microsystem.

**Improving Value in an HIV Pre-Exposure Prophylaxis Clinic for Veterans**

This project focused on reducing unnecessary sexually transmitted infection testing in an HIV pre-exposure prophylaxis clinic for veterans. At baseline, the clinic screening rate averaged 1.2 rectal or pharyngeal tests per patient visit. Detection rates of chlamydia and gonorrhea were low, at 2.3% and 5.0% respectively. The cost associated with testing was $63,000 annually. The team aimed to reduce testing by 10% while maintaining or increasing detection rates. The students observed the clinic’s operations and compared them to a best practice clinic and to CDC guidelines. They also interviewed and surveyed the clinic’s medical director, medicine residents, a nurse practitioner, pharmacist, psychotherapist, nurse manager, vocational nurse, and data analyst. They learned that patients were tested without a risk assessment and that clinicians did not have adequate knowledge to conduct risks assessments. In response to these findings, the team implemented a clinician education intervention, along with an electronic health record template for risk assessment, and later performed audits for use of the template. The template was ultimately used in 80% of encounters. Testing decreased by 23% while detection rates stayed the same (for gonorrhea) or increased (for chlamydia). This resulted in savings of approximately $14,000 annually. Students learned that making a change requires understanding stakeholders’ different perspectives, responsibilities of different clinic staff, and current protocol and procedures. The clinical team noted “a culture change toward more risk-based screening.”

**Improving Hypertension Control in Black Patients at an Academic Primary Care Clinic**

This project focused on reducing a health care disparity in hypertension control in a primary care clinic. At baseline, the rate of uncontrolled hypertension among Black patients in this primary care clinic was significantly higher (35%) than that of patients of all other races (26%). The student team set a goal of closing this gap and reducing the rate of uncontrolled hypertension among Black patients to that of patients of all other races within six months. To explore potential reasons for this disparity, students performed a literature review, interviewed nursing staff, the clinic practice manager, and patients. They learned that the underlying causes varied from patient to patient. They designed an intervention in which a student on the team called each Black patient with uncontrolled hypertension to discuss the patient’s understanding of hypertension, hear their concerns about their ability to manage this condition, and schedule follow-up clinic appointments, if appropriate. Over the course of the project, the rate of uncontrolled hypertension among Black patients decreased from 35% to 30%, short of the team’s stated goal of 26%. However, the project increased utilization of blood pressure appointments with nurses which were often more convenient for patients. It also initiated a change across the health system to the electronic medical record so that all office visit blood pressure measurements are utilized in determining hypertension control status, not just those from primary care. At the end of the students’ curricular time, the effort was taken over by clinic staff and the scope increased to encompass all primary care clinics within the academic medical center. The clinic QI lead reported that this project improved their microsystem, commenting that “embedded telephone outreach and follow-up workflow is now being developed, in large part to the enthusiasm and results from the students’ intervention.” Additionally, the students achieved learning goals in the areas of quality improvement and health disparities.
