# Supplementary material for: Bridging medical education goals and health system outcomes: An instrumental case study of pre-clerkship students’ improvement projects
Source: Perspect Med Educ. 2022 Apr 8;11(4):179–86. doi: 10.1007/s40037-022-00711-1 (PMC9391531; doi:10.1007/s40037-022-00711-1)
Supplement: Supplementary file 3 — Resource 3. Survey of faculty coaches and QI leads to follow up on Clinical Microsystem Clerkship (CMC) health systems improvement project status [file 40037_2022_711_MOESM3_ESM.docx]

ESR 3. Survey of faculty coaches and QI leads to follow up on Clinical Microsystem Clerkship (CMC) health systems improvement project status

NOTE: This survey was administered through Qualtrics

Please check the box if you consent to participate in this study.

- Yes, I consent to participate in CMC HSI project follow up study (link to information sheet for IRB #19-27272 provided)
- No (Skip to end of survey)

**Please complete the survey for each CMC project you coached or participated in**

Select Your Name: (list of coaches and QI leads)

Select Project: (list of CMC HSI projects)

Was another QI lead (i.e., not you) significantly involved in this project? If so, please provide their name(s).

- Yes ________________________________________________
- No

Please rate your confidence in your ability to lead a QI project:

|  | Strongly disagree | Somewhat disagree | Neither agree nor disagree | Somewhat agree | Strongly agree |
| --- | --- | --- | --- | --- | --- |
| Prior to completing this QI project I felt confident in my ability to lead a QI project |  |  |  |  |  |
| After completing this QI project, I felt confident in my ability to lead a QI project |  |  |  |  |  |

What change(s), if any, occurred in the microsystem or health system because of the CMC project?

________________________________________________________________

________________________________________________________________

How would you characterize the impact of the project on the microsystem at the time when the students left to start in F2 (approximately in November 2018)?

- None (explain) ________________________________________________
- Minimal (explain) ________________________________________________
- Moderate (explain) ________________________________________________
- Substantial (explain) ________________________________________________

Did the project have a lasting impact after the students left the microsystem?

- Yes
- No

*If YES:* You answered that the project **DID** have a lasting impact after the students left the microsystem, which best describes the reason why? (Please check all that apply)

- Project (including all, some piece of it, or next phase) was taken on by a new cohort of students
- Project (including all, some piece of it, or next phase) was taken on by health system staff
- Project has been 'hardwired' into the microsystem, requires minimal effort to sustain
- Other, please explain ________________________________________________

*If NO:* You answered that the project did **NOT** have a lasting impact after the students left the microsystem.  Which of the following describes the reason(s) why not?

(Please check all that apply)

- Project never really got off the ground
- Project identified other areas that need to be prioritized
- Project was no longer relevant (circumstances/priorities changed)
- Once students left there was no one to do the work
- Other, please explain ______________________________

Please briefly explain what factors contributed to the current status of the project.

________________________________________________________________

________________________________________________________________

________________________________________________________________

________________________________________________________________

Overall, this project was a success for:

|  | Strongly disagree | Somewhat disagree | Neither agree nor disagree | Somewhat agree | Strongly agree |
| --- | --- | --- | --- | --- | --- |
| improving the microsystem |  |  |  |  |  |
| students' understanding of the principles of health systems improvement |  |  |  |  |  |

Please explain why you agree/disagree with each of the above:

________________________________________________________________

________________________________________________________________

________________________________________________________________

I have completed the survey for all projects I coached in 2017-18.

- Yes
- No
