# Supplementary material for: Bridging medical education goals and health system outcomes: An instrumental case study of pre-clerkship students’ improvement projects
Source: Perspect Med Educ. 2022 Apr 8;11(4):179–86. doi: 10.1007/s40037-022-00711-1 (PMC9391531; doi:10.1007/s40037-022-00711-1)
Supplement: Supplementary file 5 — Resource 5. List of 2017–18 University of California, San Francisco Clinical Microsystem Clerkship health system improvement projects, organized by clinical setting and specialty [file 40037_2022_711_MOESM5_ESM.docx]

**ESR 5. List of 2017-18 University of California, San Francisco Clinical Microsystem Clerkship (CMC) Health System Improvement projects, organized by clinical setting and specialty.**

| **Clinical Setting** | **CMC Health Systems Improvement Project Topics** |
| --- | --- |
|  |  |
| **Inpatient Care** |  |
| Emergency Medicine | Door To Needle Time |
| Emergency Medicine | Sepsis Bundle Compliance |
| Adult Medicine | Delirium Reduction |
| Adult Medicine | Mobility Improvement |
| Adult Medicine | Fall Reduction |
| Adult Medicine | Sleep in the Hospital |
| Adult Medicine | Discharge Medication Reconciliation |
| Adult Medicine | Interhospital Transfer Redesign |
| Adult Medicine | Patient Care Experience in the Safety Net |
| Adult Medicine | Pre-Exposure Prophylaxis (PrEP) Referrals |
| Adult Medicine | Naloxone Prescribing at Discharge |
| Adult Medicine | Palliative Care Patient Satisfaction |
| Pediatrics | Delirium Reduction in Critical Care |
| Pediatrics | Respiratory Distress in Asthma |
| Psychiatry | Psychiatry Readmissions Reduction |
| Psychiatry | Tobacco Use Assessment and Counseling |
| Nursing Home | Chronic Pain Treatment in Older Adults |
| Nursing Home | Urinary Tract Infection Work-Up and Prevention |
|  |  |
| **Ambulatory Care** |  |
| Primary Care | Flu Vaccination |
| Primary Care | Hypertension Control in Black Patients |
| Primary Care | Hypertension Management |
| Primary Care | Polypharmacy Among Older Patients |
| Primary Care | Depression and Suicidality |
| Primary Care | Interprofessional Veteran Transgender Clinic |
| Primary Care | Hepatitis C Treatment Rates |
| Primary Care | Hepatocellular Carcinoma Screening |
| Primary Care | Alcohol Use Disorder in Veterans |
| Primary Care | Sexual Therapy Referral at a Women's Health Clinic |
| Primary Care | Interpersonal Violence Education and Screening |
| Primary Care | Depression Treatment |
| Primary Care | Depression Screening |
| Primary Care | Cycle Time Reduction in Women's Health |
| Primary Care | Naloxone and Opioid Overdose |
| Infectious Diseases | Improving Value in HIV PrEP Clinic |
| Neurology | Vaccinations in Multiple Sclerosis |
| Neurology | Flu Shots in the Pediatric Brain Center |
| Neurology | Neurogenic Bladder |
| Psychiatry | Mental Health Care for Veterans |
| Psychiatry | Non-Emergent Mental Health Care |
| Pediatrics | Patient Wait Time |
| Pediatrics | Secondhand Smoking Cessation |
| Pediatrics | Sexually Transmitted Infection Screening |
| Rheumatology | High-Risk Immunosuppressive Medication Administration |
| Rheumatology | Safe Prescribing of Hydroxychloroquine |
|  |  |
| **Perioperative Care** |  |
| Ob-Gyn | Pregnancy-Related Deaths from Obstetric Hemorrhage |
| Ob-Gyn | Time from Decision to Incision in Unplanned Cesarean Sections |
| Ob-Gyn | Enhanced Recovery Pathways in Minimally Invasive Gynecology Oncology Surgery |
| Anesthesia | Multimodal Analgesia Use in Minimally Invasive Gynecology Oncology Surgery |
| Surgery | Post-Lung-Transplant New Onset Diabetes |
| Surgery | Post Lung Transplant Caregiver Wellness |
| Surgery | A Surgery Wellness Program for Frail Patients |
| Surgery | Post-Endocrine Surgery Readmission Reduction |
|  |  |
| **Other** |  |
| Clinical Informatics | An Application Testing Environment for an Electronic Health Record |
