# Supplementary material for: Bridging medical education goals and health system outcomes: An instrumental case study of pre-clerkship students’ improvement projects
Source: Perspect Med Educ. 2022 Apr 8;11(4):179–86. doi: 10.1007/s40037-022-00711-1 (PMC9391531; doi:10.1007/s40037-022-00711-1)
Supplement: Supplementary file 6 — Box. Sample project descriptions [file 40037_2022_711_MOESM6_ESM.docx]

**Box. Two sample project descriptions**

**Reducing Pregnancy-Related Deaths from Obstetric Hemorrhage at a County Hospital**

This project focused on reducing obstetric mortality by implementing a new, state-wide, institutional performance metric at a County Hospital. This project aimed to implement an evidence-based, quantitative blood loss (QBL) measurement process for 80% or more births each year (the state-wide benchmark) from a current baseline of zero. Based on a literature review, interviews, focus groups, and surveys of midwives, nurses and physicians, students identified the lack of protocols, training, and necessary equipment as important barriers to address. They partnered with quality improvement leaders to develop a standard protocol for QBL assessment, delivered an in-person and online training for providers, and obtained a dedicated scale to facilitate measurement of QBL by weighing linens. After implementation, the team performed audits of births and provided feedback to reinforce the importance of QBL assessment. This team exceeded the project aim, achieving average weekly compliance with QBL techniques in 84% of deliveries within nine months. Surveys of clinic staff at the conclusion of the project characterized the degree of improvement as “substantial,” commenting that “[the] blood loss calculation system had been completely changed to quantitative compared to estimated” as a result of the student project. Additionally, students reported learning how to incorporate medical literature in clinical practice, the importance of understanding workflow when designing systems changes, and the need to involve all stakeholders when developing interventions. The QBL process became the new “norm” and continued after the students left the microsystem.

**Improving Value in an HIV Pre-Exposure Prophylaxis Clinic for Veterans**

This project focused on reducing unnecessary sexually transmitted infection testing in an HIV pre-exposure prophylaxis clinic for veterans. At baseline, the clinic screening rate averaged 1.2 rectal or pharyngeal tests per patient visit. Detection rates of chlamydia and gonorrhea were low, at 2.3% and 5.0% respectively. The cost associated with testing was $63,000 annually. The team aimed to reduce testing by 10% while maintaining or increasing detection rates. The students observed the clinic’s operations and compared them to a best practice clinic and to CDC guidelines. They also interviewed and surveyed the clinic’s medical director, medicine residents, a nurse practitioner, pharmacist, psychotherapist, nurse manager, vocational nurse, and data analyst. They learned that patients were tested without a risk assessment and that clinicians did not have adequate knowledge to conduct risks assessments. In response to these findings, the team implemented a clinician education intervention, along with an electronic health record template for risk assessment, and later performed audits for use of the template. The template was ultimately used in 80% of encounters. Testing decreased by 23% while detection rates stayed the same (for gonorrhea) or increased (for chlamydia). This resulted in savings of approximately $14,000 annually. Students learned that making a change requires understanding stakeholders’ different perspectives, responsibilities of different clinic staff, and current protocol and procedures. The clinical team noted “a culture change toward more risk-based screening.”
